# Healthcare system and its challenges

**DOI:** 10.4103/0253-7613.75656

**Published:** 2011-02

**Authors:** APJ Abdul Kalam

**Affiliations:** Honourable Former President of India www.abdulkalam.com

I am indeed delighted to participate in the 43^rd^ Annual Conference of Indian Pharmacological Society (IPS 2010) at Hyderabad. I am also happy to know that the Indian Council for Medical Research is celebrating its Centenary year. My greetings to all of you. While I am here with all of you, I would like to share my views on the topic “Healthcare system and its challenges”.

I will be discussing about:

- Spirit of a Healthcare provider
- Innovative Cancer Vaccine Development
- Six virtues of a health care provider
- Moral Leadership
- New vistas in the form of Pharmacovigilance
- Finally, I would be presenting how different technologies are converging and reshaping the pharma and medicine sector

## The spirit of a healthcare provider: What can I give?

Dear friends, I would like to share my experience with two medical professionals who are unique, in their areas of work and their choice of life. One doctor, whom I know very well, faced a tragedy early in his life when lost his father in his lap, because medical help could not be reached in time. In a hard way, he completed his MBBS, and became a doctor, selected a tribal area for transformation.

He successfully implemented his mission of uplifting the poor and improve their quality of life. The second medical professional is a researcher in thrombosis. He started a research institute in UK and later established a Thrombosis Research Institute in Bangalore, which I inaugurated.

In the present circumstances and environment, it was inspiring to see, how a doctor has put all his dreams in mainstreaming the tribal citizens of Karnataka for the last 25 years through Vivekananda Girijana Kalyan Kendra (VGKK), at BR Hills. When I visited BR Hills in 1998 and subsequently in 2006, I could see substantial developments in that area. I could see a “New Tribal Hospital”, roads and education environment and above all the earning capacity of the tribal citizens have been increased with the technology resource centre as a base. Dr. H. Sudarshan, is the inspiring architect of this societal transformation. The mission which he started has spread to many parts of the country including the Andaman and Nicobar islands and Arunachal Pradesh. Dr. Sudarshan and his team have been selecting difficult regions and making a difference to the people of that region by their own way of life which was started in a small hut. The nation needs thousands of Dr. Sudarshans for providing healthcare to our rural citizens. I am sure some of you may emulate Dr. Sudarshan in this noble mission


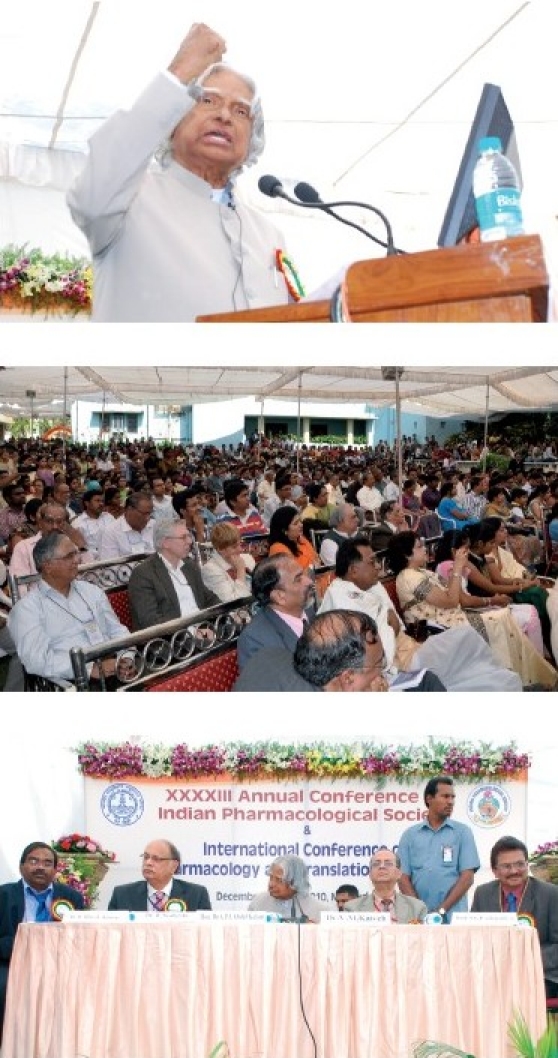


Let me now discuss the research contribution of Dr. V.V. Kakkar of Thrombosis Research Institute that I inaugurated at Bangalore on 18 Dec 2006. This is pioneered by the founder Director Dr. V.V. Kakkar based on the institution he established in UK. Based on the discussion I had with the experts in this field, let me share with you, the research content on thrombosis prevention that has a direct relevance to cardiovascular, neurological and other diseases. The knowledge obtained through the new research is likely to impact positively specialties like cardiology, neurology, peripheral vascular disease, open-heart surgery, coronary vascular interventions and artificial heart valves. Congenital gene-linked disorders like hemophilia may be benefited by new genomic medical technology. Availability of drugs like heparin opened the door for treatment such as open-heart surgery, cardiac catheterization and angiography. Thrombolytic drugs like streptokinase have reduced ICCU mortality of acute heart attacks from 15% to 5%. Appropriate and early timely use of these drugs is saving lakhs of lives all over the world. Heparin and clot-buster drugs have contributed tremendously in saving patients with deep venous thrombosis and clot embolism to lungs. Recent contributions in the management of acute brain stroke are of great clinical significance. We need newer thrombolytic drugs more ideally suited for safe and effective use in acute heart attacks and brain strokes. They should not only be more effective, but they should not have systemic side effects and the rebound thrombosis. One of the nanotechnology applications ideally suited for this purpose is drug mailing, where the injected drug will go to the site of the clot and be effective locally at that required site.

## Innovative Cancer Vaccine Development

Let me now discuss my recent experience of innovative vaccine development for cervical cancer. In April 2010, while in Louisville, USA, I met researchers from Brown Cancer Research Center who are developing a vaccine for cervical cancer which affects many women in the nation. A World Health Organization study reveals that every year 132,000 women are diagnosed with this particular kind of cancer and nearly 75,000 die from the disease. The proposed vaccine would be developed on protein drawn out of tobacco leaves and would cost nearly two dollars for every individual. I am sure, the healthcare community assembled here would like to take up the development in such areas of societal importance.

## Six virtues of a healthcare provider

When I am with the medical community, I would like to share an experience with Choakyi Nyima Rinpoche, the Chief Monk in Kathmandu and a medical researcher. After nearly a kilometer of walk, I reached the white Kumbha where the chief monk and his disciples were waiting to receive me. After receiving me, the Chief Monk said, let us go to our study room and I followed him. He climbed the first floor, the second floor, the third floor, the fourth floor and the fifth floor, just like a young boy. Probably the life style has a positive impact on the mind and body. All along I was following and following. When I reached his chamber, I saw a laboratory in a spiritual environment over looking the Himalayas. What surprised me was, his research students come from different parts of the country. Particularly he introduced me to his co-author David R Shlim, MD, who is working on a research area “Medicine and Compassion”. The Chief Monk Choakyi Nyima Rinpoche and myself exchanged few books. The Monk has written with Dr. David R. Shlim a book titled “Medicine and Compassion”. I liked this book and read it during my journey from Kathmandu to Delhi. This book gives six important virtues which a medical practitioner has to possess towards their patients.

First virtue is generosity; the second virtue is pure ethics; third is tolerance, fourth is perseverance, fifth is cultivating pure concentration and the sixth virtue is to be intelligent. These virtues will empower the care givers with a humane heart.

## Moral leadership

Moral leadership involves two aspects. First, it requires the ability to have compelling and powerful dreams or vision of human betterment. Moral leadership requires a disposition to do the right thing and influence others also to do the right things. There is a perception that a certain number of pharmaceutical products sold in India are counterfeit or of substandard quality. These drugs are generally made by unscrupulous elements and supplied surreptitiously to chemist shops through illegal channels. The pharmacological education has an important role to play in equipping the professionals with the knowledge and ability to detect the entry of unauthorized drugs into circulation. Also, every effort should be made to check the manufacture, sale and distribution of spurious drugs. The Central and State Governments have to ensure that the Drugs and Cosmetics Act is properly enforced to check this nefarious practice. Healthcare professionals must ensure the source of supply of drugs is reliable to check the menace of spurious drugs.

On the other hand, moral leadership will also require you to be dedicated to the welfare of the most important element of healthcare – the patient. You must constantly keep his or her benefit in the mind as removing the pain is your ultimate aim. For that, you will have to take a holistic outlook at pharmacological services being provided – keeping in mind the side effects of the drugs which are seen in the long run.

Last week, I came across an article about how anti-cholesterol drugs can cause depression. The article mentions about the research by Indian scientists from the Center for Cellular and Molecular Biology which found that “statins” class of drugs, used to lower cholesterol, by inhibiting a key enzyme responsible for its bio-synthesis in the body can also cause the side effect of depression and anxiety. The scientists have shown that chronic cholesterol depletion by statins impairs the function of the receptors for serotonin, a neuro-transmitter in the brain that controls mood and behavior. Maintaining normal cholesterol levels is important for the function of cell membrane receptors for serotonin. The latest study shows that cholesterol depletion in the brain affects the function of serotonin receptors leading to depression and anxiety. So dear friends, as healthcare missionaries, you will have to deal and evolve yourself to handle such complex interlinking of the human body and drug effect.

## Pharmacovigilance

Pharmacovigilance is the pharmacological science relating to the detection, assessment, understanding and prevention of adverse effects, particularly long term and short term side effects of medicines. With the increasing number of new medicines being introduced regularly in the nation, the new patent regime which makes our pharmaceutical firms to discover and develop new medicines and the phenomenal growth in the clinical research in India, there is a need for enhanced focus on the domain of Pharmacovigilance to clearly identify the side effects of new medicines and establish strategies to counter them. Pharmacovigilance will not only effect drug development but also be a critical input for the policy making domain.

My expert friends suggest that the ready availability of safer and more effective medicines of good quality inspires confidence and trust among patients. Pharmaco-vigilance is an essential part of the public programmes that underpin the reliable availability of sound medicines and it needs to be understood, supported and promoted at the highest levels.

To achieve this, it is necessary that information about drug safety programmes to be easily available to the public so that the central role of the patient in the rational and safe use of medicines is understood. The public has in recent years increasingly influenced health professionals’ prescribing and patterns of drug use. This influence and greater awareness on the part of the public is attributable in part to the role of the media and Internet. High expectations of all service providers and medical institutions have developed.

Pharmacovigilance has four dimensions – detection, deduction, decision and dissemination. Detection is done through certain experimental techniques and would employ a large number of observational methods. This would require the creation and development of scientific centers and trained workforce in this dimension. Existing mechanisms of communication have to be improved for a better feedback mechanism and effective flow of observations. Deduction would need the analysis of the available information and hence the creation of a wide and secure data scanning and reporting mechanism with monitoring systems. Similarly, a robust and transparent system needs to be established for the decision and dissemination of the results with an action focused approach with involves the private, public and community sectors. Can the experts assembled here evolve such a comprehensive strategy for realizing a robust pharmacovigilance system in the nation?

## Conclusion: Post-Genomic Health Care

In conclusion let me discuss how the boundaries between different forms of technologies are vanishing and how this will impact the future of pharmacological domain.

Friends, if the last fifty years belonged to information technology, there is no doubt that the next fifty years will belong to biotechnology in combination with information and nanotechnology. In biology, undoubtedly one defining moment came in February 2001, when the draft human genome was published simultaneously by two different research groups. By now we all know that the human genome consists of about 3.3 billion nucleotides, but two human beings differ in barely 0.1% of the genome. And yet many of the individual variations seem to be explainable by precisely this tiny variation. So now it is actually possible to speak confidently about “personal medicine” whereby it is possible to fine-tune drug dosages and treatment regimens to individual variations. But all this requires completely new science and technology that combines IT and BT.

When the project to sequence the human genome was announced in the mid-1990s, everyone thought that it would be an impossible task. As it happened, the first human genome sequence took about ten years and cost about $ 10 billion. The cost keeps dropping by a factor of about ten every two years. Already there is a company called Complete Genomics that promises to deliver a complete human genome for $5,000 by this year (2010). If you don’t want the full genome but just want to check for a few bio-markers that may cause you trouble in future, that kind of information is available for just $ 299 even today! In the recent Indian industries fare, one of the genome lab of CSIR was doing free of cost genome marker for few individuals. So, the genome technology for characterizing in the cost effective way, the individual genes are closer and closer.

One of the most important aspects of drug delivery is called the “formulation”. Basically the formulation is nothing more than “the pill” – that is, the coating of a drug with bio-compatible material, so as to regulate the rate at which the drug is released into the body. Until now the design of formulations has been a hit and miss affair. But with the use of nano-technology, it becomes possible to design quite precise ways of releasing the drug. For example, until a few years ago, insulin could only be injected in most cases. But now it is possible to inhale insulin. The medical community is working on glycogen-like-proteins, that will do the job of insulin and can be taken orally, and that is one of the most promising approaches. The systematic design of the delivery mechanism, or the “formulation”, requires a deep understanding of how the body acts on the pill, and how the drug acts on the body.

I was reading an article on personalized medicine in the magazine American Scientist (issue of September 2010). It talks about how medical science is now seeing a spontaneous action due to the completion of human genome project. It says, that in the times ahead, the role of one-size-fit-all medicines will now be replaced by medicine tailored to individuals. Specific regions will be able to assemble their customized healthcare solutions depending on the inputs of custom, climate and an individual. In the near future an eco- system of healthcare and software providers will empower doctors to treat each patient as a unique individual and stem cells can be fashioned into treatments. Individual genomes will get sequenced every year to check for disease and abnormalities and customized treatment will be delivered and “shaping an individual biology will be a part of life”. This is the future, of pharma and medicine, which all of us is looking forward to.

With these words, let me congratulate the Indian Pharmacological Society for promoting need-based research in pharmacology and other allied sciences. My best wishes for success in all their missions.

May God Bless You.

